# Validation Study to Determine the Accuracy of Widespread Promoterless EGFP Reporter at Assessing CRISPR/Cas9-Mediated Homology Directed Repair

**DOI:** 10.3390/cimb44040116

**Published:** 2022-04-12

**Authors:** Wanqing Xu, Qingxia Zuo, Dongyan Feng, Changsheng He, Cailing Lin, Dongchao Huang, Yanbin Wan, Feng Chen, Guosheng Mo, Qi Sun, Hongli Du, Lizhen Huang

**Affiliations:** School of Biology and Biological Engineering, South China University of Technology, Guangzhou 510006, China; 201821037224@mail.scut.edu.cn (W.X.); 201920146246@mail.scut.edu.cn (Q.Z.); 202021049642@mail.scut.edu.cn (D.F.); 201920146224@mail.scut.edu.cn (C.H.); 202020148465@mail.scut.edu.cn (C.L.); 202120149733@mail.scut.edu.cn (D.H.); 202121050517@mail.scut.edu.cn (Y.W.); 202120149291@mail.scut.edu.cn (F.C.); 201830881184@mail.scut.edu.cn (G.M.); 201930501364@mail.scut.edu.cn (Q.S.); hldu@scut.edu.cn (H.D.)

**Keywords:** CRISPR/Cas9, homology-directed repair, precise gene editing, random integration, promoterless EGFP reporter

## Abstract

An accurate visual reporter system to assess homology-directed repair (HDR) is a key prerequisite for evaluating the efficiency of Cas9-mediated precise gene editing. Herein, we tested the utility of the widespread promoterless EGFP reporter to assess the efficiency of CRISPR/Cas9-mediated homologous recombination by fluorescence expression. We firstly established a promoterless EGFP reporter donor targeting the porcine *GAPDH* locus to study CRISPR/Cas9-mediated homologous recombination in porcine cells. Curiously, EGFP was expressed at unexpectedly high levels from the promoterless donor in porcine cells, with or without Cas9/sgRNA. Even higher EGFP expression was detected in human cells and those of other species when the porcine donor was transfected alone. Therefore, EGFP could be expressed at certain level in various cells transfected with the promoterless EGFP reporter alone, making it a low-resolution reporter for measuring Cas9-mediated HDR events. In summary, the widespread promoterless EGFP reporter could not be an ideal measurement for HDR screening and there is an urgent need to develop a more reliable, high-resolution HDR screening system to better explore strategies of increasing the efficiency of Cas9-mediated HDR in mammalian cells.

## 1. Introduction

The CRISPR/Cas9 system has recently gained popularity as a means of performing gene editing across many fields. In this system, single guide (sg)RNAs are used to target Cas9 protein to induce on-target DNA double strand breaks (DSBs); gene editing is then accomplished through different DSB repair pathways, such as non-homologous end joining (NHEJ), microhomology-mediated end joining (MMEJ), and homologous recombination (HR) [1]. Among them, the homology-directed repair (HDR) pathway has been widely used to introduce precise genetic modifications [2]. Through artificial delivery of exogenous DNA templates with homology to the region around the DSB, HDR can achieve precise target gene editing, such as introducing a specific base substitution or the insertion/deletion of site-specific DNA sequences. Precise editing allows interrogation of the functionalities of any piece of DNA in the genome of any species for many biomedical purposes [3].

However, HDR-mediated precise editing is inefficient because HDR is predominantly restricted to the S/G2 phases of the cell cycle and occurs less frequently than NHEJ [4,5]. Accordingly, various approaches to bias DSB repair in favor of HDR have been proposed to increase the efficiency of precise editing, including modifications to Cas9 or sgRNAs to mediate an attachment between the donor and CRISPR components [6,7,8,9,10,11]. Moreover, altering the construction of exogenous DNA templates has also been applied to make templates more stable [12]. In addition, cell cycle regulation involved in blocking NHEJ/promoting HDR or restrict SpCas9 activity to particular phases of the cell cycle have also been applied [13,14,15,16,17].

In addition to these aspects, establishing an accurate and intuitive system of evaluating HDR is a key prerequisite of monitoring the efficiency of Cas9-mediated precision editing. Currently, there are two common HDR reporter systems: (1) using exogenous DNA as the targeted sites for intentional genetic modification, in which exogenous sequences are integrated into the genome or expressed transiently. As examples, Yan et al. developed a surrogate reporter plasmid with self-cleavage and repair ability during CRISPR/Cas9-induced HDR events [18]. Cai et al. constructed transgenic HEK 293FT cells that stably express blue fluorescent protein (BFP); HDR efficiency is evaluated by the transformation of BFP to EGFP [8]. However, the most widely used HDR reporter system is (2) precise insertion of a promoterless reporter gene into the endogenous locus, with *GAPDH* being the most universal target site because of its high and stable expression in different cell lines.

Yu et al. evaluated the effect of chemical modification of the 5′-end of exogenous DNA donors on Cas9-mediated precise editing by applying the EGFP promoterless reporter system targeting *GAPDH* [12]. Knock-in (KI) efficiency was measured by flow cytometry and it was demonstrated that modifying 5′-end of the donor could lead to a 5-fold increase in KI rates at various genomic loci in human cancer and stem cells. Li et al. also applied a similar promoterless EGFP reporter to access the HDR efficiency of a modified Cas9/donor system, in which Cas9 was fused with a transcription factor DNA-binding domain, and then the corresponding DNA sequence bound by the transcription factor was appended to the ends of donor sequences to colocalize donor and CRISPR components [7]. The promoterless EGFP reporter involved a T2A–EGFP coding sequence (CDS) targeting the 3′-end of *GAPDH*. HDR efficiency was accessed by measuring EGFP fluorescence via flow cytometry and EGFP protein levels via Western blot. Up to a 6-fold increase in the KI rate was demonstrated by combining the modified Cas9/donor with compounds that promote HDR. Additionally, promoterless EGFP reporters targeting other endogenous loci have been used to evaluate the HDR efficiency of different strategies in different cells (Table S1) [18,19,20,21,22,23,24].

To study precise editing in porcine cells, we established a promoterless EGFP reporter targeting the porcine *GAPDH* locus. Theoretically, EGFP expressed only when the EGFP reporter gene has been specifically inserted into *GAPDH* by HDR. Before studying precise editing in this system, we strictly evaluated the accuracy of established GFP reporter systems in porcine cells. Curiously, EGFP expression from the promoterless donor template was observed to be at unexpectedly high levels in porcine cells, with or without Cas9/sgRNA. When the *GAPDH*-targeting porcine reporter was transfected alone, even higher EGFP expression was detected in human cells and those of other species (HepG2, HepaRG, 293T, and CHO-K1). These data suggest that the EGFP reporter was not exclusively expressed in CRISPR/Cas9-induced HDR cells, which brings into question the accuracy of this reporter system.

## 2. Materials and Methods

### 2.1. Cell Culture

Porcine fetal fibroblasts (PFF) cells (Chinese academy of sciences, Guangzhou, Guangdong province, China) were maintained in DMEM (C11995500BT, Thermo Fisher Scientific, Waltham, MA, USA) supplemented with 15% Fetal Bovine Serum (FBS) (26010074, Thermo Fisher Scientific, Waltham, MA, USA), 1% penicillin/streptomycin (15140122, Thermo Fisher Scientific, Waltham, MA, USA), 1% non-essential amino acids (11140050, Thermo Fisher Scientific, Waltham, MA, USA), and 1% GlutaMAX (35050061, Thermo Fisher Scientific, Waltham, MA, USA); PK15 cells (CCL-33, ATCC, Manassas, VA, USA) were maintained in MEM (C11095500BT, Thermo Fisher Scientific, Waltham, MA, USA) supplemented with 10% FBS and 1% penicillin/streptomycin. CHO-K1 (CCL-61, ATCC, Manassas, VA, USA) cells were cultured in DMEM/F-12 (11320033, Thermo Fisher Scientific, Waltham, MA, USA) supplemented with 10% FBS, 100 U/mL penicillin and 100 μg/mL streptomycin; HepaRG cells (MMHPR116, Millpore, Burlington, MA, USA) were cultured in William’s E Medium (12551032, Thermo Fisher Scientific, Waltham, MA, USA) supplemented with 10% FBS, and 1% penicillin/streptomycin; HepG2 (HB-8065, ATCC, Manassas, VA, USA) and HEK293T (CRL-3216, ATCC, Manassas, VA, USA) were maintained in DMEM supplemented with 10% FBS, and 1% penicillin/streptomycin. All cell lines were maintained at 37 °C in a humidified incubator with 5% CO_2_.

### 2.2. Design and Construction of the Promoterless EGFP Reporter System to Assess CRISPR/Cas9—Mediated HDR

The HDR screening system here included a promoterless EGFP reporter that could be specifically knocked into endogenous *GAPDH* loci by the CRISPR/Cas9 mediated-HDR. Successful tagging enabled the HDR efficiencies to be determined by measuring the EGFP fluorescence intensity. 

pCMV-Cas9 vector, which has a selectable neomycin marker, was obtained from Addgene (41815, Addgene, Watertown, MA, USA). sgRNAs were designed near the stop codon of *GAPDH* by N20NGG rule. Their on-targets efficiencies and off-targets potencies were predicted and scored by CRISPOR (http://crispor.tefor.net, accessed on 2 August 2021) [25,26]. Selected sgRNAs sequences were listed in Table S2. Additionally, ForeCast [27], inDelphi [28], and Lindel [29] were used to predict the indel signatures mediated by different sgRNAs. The sgRNA expression vector was constructed as previously described [30,31]. Briefly, oligonucleotides were annealed to form double-stranded DNA, and then cloned into BbsI (R0539S, New England Biolabs, County Road, Lpswich, UK)—digested U6-sgRNA vector. The construction strategies were listed in Table S3. PFF cells were electroporated with candidate sgRNAs and pCMV-Cas9. Cells were harvested after 48 h and the target sites were amplified for Sanger sequencing. Then the targeting efficiency of sgRNA was evaluated by TIDE (http://tide.nki.nl/, accessed on 17 August 2021) [32].

**Figure 1:**
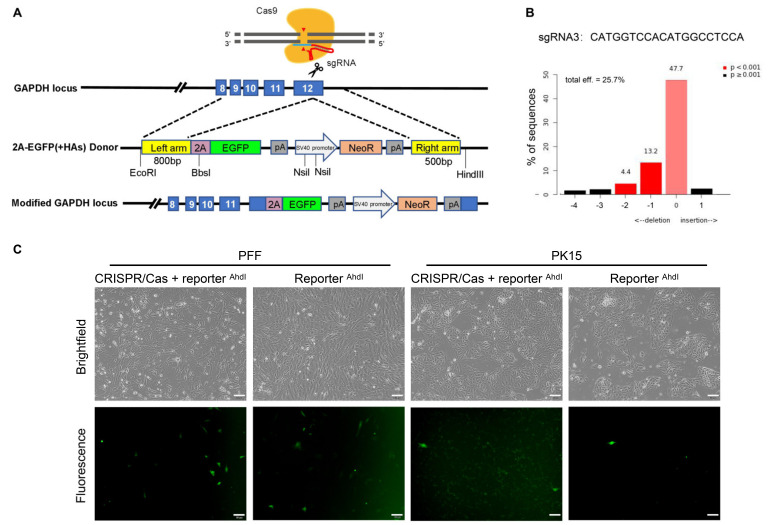
Overview of HDR reporter targeting the *GAPDH* locus. (**A**) Schematic of *GAPDH* targeted HR reporter strategy. The CRISPR/Cas9 targeting the stop codon (TAA) of *GAPDH* exon 12. Reporter vector was designed containing an 800-bp left homologous arm cover the exon 8, 9, 10, 11 and 12 upstream from the stop codon, and a 500-bp right homologous arm downstream of the stop codon. (**B**) Validation intracellular indels efficiency and indels pattern of sgRNA3 targeting exon 12 of porcine *GAPDH* by TIDE analysis. Cells were transfected with CRISPR/Cas9 system and harvested after 48 h. (**C**) Fluorescence microscope of PFF and PK15 cells transfected with CRISPR/Cas9 system and reporter digested with AhdI (Reporter ^AhdI^) or Reporter ^AhdI^ only 48 h post transfection. Scale bar: 50 μm.

For the p2A-EGFP (+HAs) donor, homologous arms were designed both upstream and downstream of the termination codon TAA site (Figure 1A). The 800-bp left HA and 500-bp right HA were amplified by genomic PCR and cloned into the pUC57 vector. The entire *EGFP* coding sequence and *NeoR* were amplified from the pEGFP-N2 and pcDNA3.3-hCas9 vectors respectively. Subsequently, the EGFP sequence was ligated with P2A peptide and then inserted right between the left and right arms with *NeoR*. Meanwhile, a PAM-altering synonymous point mutation was introduced in the reporter to improve HDR efficiency [33]. All constructs were confirmed by Sanger sequencing (BGI, Guangzhou, China). Plasmids were purified using an Endo-free Plasmid Mini kit (D6950, Omega Biotech, Norcross, GA, USA). 

### 2.3. Assessing the Efficiency of Cas9-Mediated HDR in Porcine Cells Using the Promoterless EGFP Donor

1 × 10^5^ PFF cells were harvested and resuspended in 10 μL electroporation buffer (MPK5000S, Invitrogen, Carlsbad, CA, USA) supplemented with hCas9, sgRNA, and reporter (molar ratio of Cas9:sgRNA:donor was 1:1:2, with a total of 2 μg). Reporter was digested with AhdI (R0584S, New England Biolabs) before transfection. The mixture was transfected into PFF cells using the Neon™ Transfection System (MPK5000, Invitrogen), with 1350 V/30 ms and 1 pulse. Subsequently, transfected cells were transferred to 12-well plates (3513, Corning, Corning, NY, USA), and EGFP expression was observed 48-h after transfection using fluorescence microscopy (Olympus, Shinjuku, Tokyo, Japan).

3 × 10^5^ PK15 cells were seeded into 12-well plates and reached 70–80% confluency at the time of transfection. 750 ng DNA (molar ratio of Cas9:sgRNA:reporter was 1:1:2) was transfected using 2.25 μL of GenJet™ In Vitro DNA Transfection Reagent (SL100489, SignaGen, Rockville, MD, USA). EGFP expression was observed 48 h post-transfection using fluorescence microscopy.

### 2.4. Evaluating the Promoterless EGFP Reporter in Porcine and Non-Porcine Cells

To validate the specificity of the promoterless EGFP reporter, porcine cells (PFF, PK15) and non-porcine cells (HepaRG, HepG2, HEK293T, and CHO-K1) were selected and transfected merely with the promoterless EGFP reporter.

For PFF cells, approximately 1 × 10^5^ cells were resuspended in 10 μL electroporation buffer supplemented with 2 μg reporter. Transfection was performed according to that previously described. All other cells were transfected using GenJet™ In Vitro DNA Transfection Reagent. 24 h before transfection, cells were seeded into 24-well plates (3524, Corning). When cells reached 70–80% confluency, they were transfected with 500 ng reporter using 1.5 μL GenJet™ Reagent. pEGFP-N2 was transfected in each cell group as a control. EGFP expression was observed using fluorescence microscopy and then analyzed by flow cytometry. For HEK293T cells, EGFP observation continued for 10 days after transfection.

### 2.5. Flow Cytometry

Cells were harvested 48 h post-transfection and resuspended in PBS; EGFP-positive cells were detected using a BD Accuri™ C6 plus flow cytometer. Under high-throughput mode, 10,000 events gated by FSC-H and FSC-W were collected, which contained most non-aggregated live cells. Data from FITC channels were extracted as EGFP. Flow cytometry data were analyzed using FlowJo 10.6.2 software (Tree Star).

### 2.6. Molecular Analysis of the Targeted Loci

To further explore the HDR events among the GFP positive cells, PCR was applied to analyze the integration status of the targeted loci. Genomic DNA from 293T in experimental and control group were isolated using the MiniBEST Universal Genomic DNA Extraction kit and then subjected to PCR analysis. 

Targeted integration was analyzed with primers *GAPDH*-F1/R1. Forward primer was designed from sequence outside the homologous regions between reporter’s left arm and human *GAPDH*, reverse primer was designed complementary to EGFP. The primers were as follows: *GAPDH*-F1: CTGAGGCTCCCACCTTTCTC, R1: TTCAGGGTCAGCTTGCCGTA. Random integration was analyzed with primers flanking the EGFP and *NeoR* sequences of the reporter. The specific primers were as follows: *GAPDH*-F2: TGCCCGACAACCACTACCTG, R2: GGCATCAGAGCAGCCGATTG; The amplified fragment with forward primer from exon 8 and reverse primer from exon 9 of human *GAPDH* locus were taken as control. The primers sequences were as follows: *GAPDH*-F3: TCAACGACCACTTTGTCAAGC, R3: GGAGAACATACCAGGTCCCTCC.

Genomic DNA from 293T cells was amplified with PrimeSTAR (R045Q, TakaRa, Kusatsu, Shiga, Japan), and the PCR conditions were as follows: 94 °C for 5 min; 30 cycles of 94 °C for 30 s, 61 °C for 30 s and 72 °C for 1 min; 72 °C for 7 min; finally terminating at 16 °C. PCR products were analyzed by gel electrophoresis. 

### 2.7. Predicting Transcription Factor Binding Sites in Different Species

The Promo databases (ALGGEN-PROMO [upc.es]) were chosen for the subsequent analysis of potential transcription factor binding sites. The homologous arm sequences were input into the database and then the corresponding transcription factor binding sites were predicted with three species (*Homo sapiens*, *Hamster,* and *Sus scrofa*).

## 3. Results

### 3.1. Successful Establishment of the Promoterless EGFP Reporter System and sgRNA Targeting the Porcine GAPDH Locus

To investigate Cas9-associated HDR events in porcine cells, we developed this HDR screening system allowing identification of HDR events through fluorescence detection. The promoterless reporter vector contained three components: HAs for porcine *GAPDH*, a *NeoR* gene expression cassette, and the *EGFP* CDS without promoter (Figure 1A and Figure S1A).

To insert the promoterless reporter immediately downstream of porcine CDS, three sgRNAs were selected based on predicted efficiency and off-target potency (Figure S1C). 48 h post-transfection in PFF cells, the Sanger sequencing results showed that significant multiple peaks appeared near the Cas9 cleavage site for all three sgRNAs, confirming the activity of the designed sgRNAs. It is noteworthy that three sgRNAs exhibited similar efficiencies (Figure 1B and Figure S1D). Meanwhile, a MMEJ-dominant indel pattern (deletions ≥ 3 bp resulting from MMEJ) can be used to select sgRNAs for optimal HDR editing [34]. Based on the prediction of indel signatures by ForeCasT, inDelphi, and Lindel (Figure S2), sgRNA3 was selected for its high MMEJ-dominant indels pattern.

### 3.2. Unexpected EGFP Expression in Porcine Cells Transfected with the Promoterless EGFP Donor and Cas9/sgRNA

Theoretically, when cells are co-transfected with the promoterless EGFP donor and Cas9/sgRNA, the 2A-EGFP will be precisely inserted into *GAPDH* loci if successful HDR occurs; Thus, HDR efficiency can be monitored by EGFP expression. Strikingly, the cells merely transfected with linearized reporter showed the similar EGFP expression level as the cells transfected with the Cas9/sgRNA and reporter. This phenomenon was observed in both PFF and PK15 cells (Figure 1C). This unexpected EGFP expression without specific DSB in the target sequence would confuse the assessment of CRISPR/Cas9-mediated HDR efficiency.

**Figure 2:**
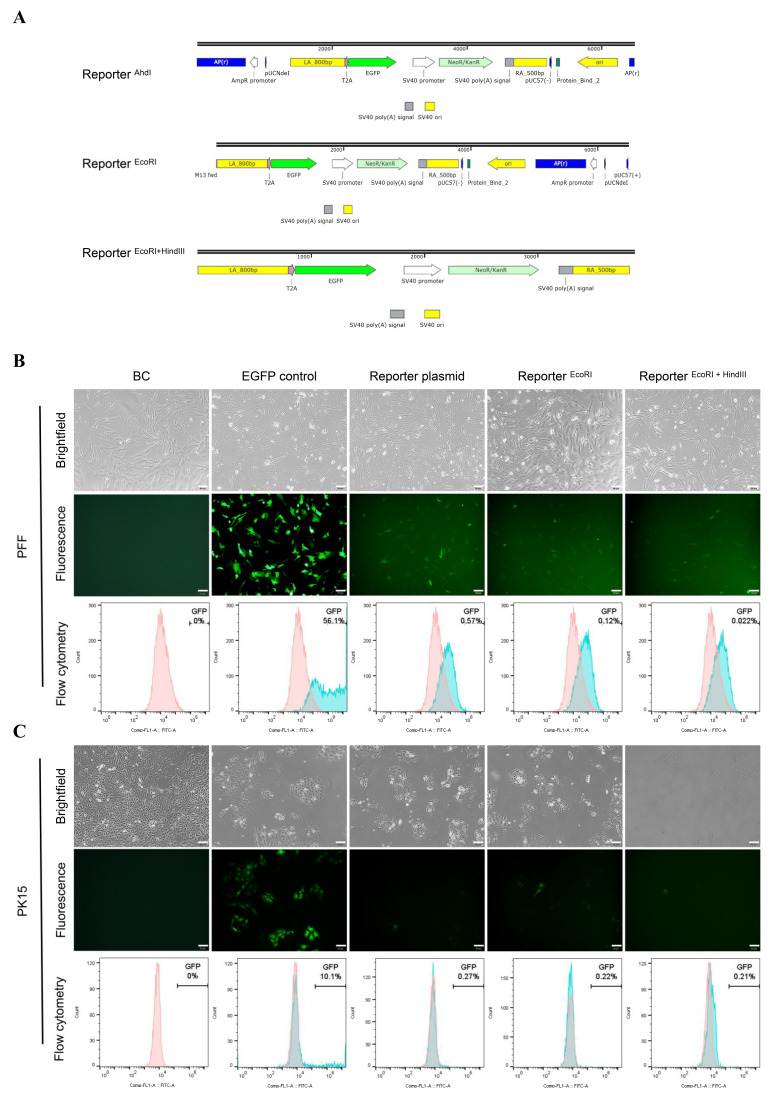
Porcine cells transfected with different forms of reporter. (**A**) Structure of the reporter digested with AhdI, EcoRI, EcoRI+HindIII, respectively. (**B**) PFF and (**C**) PK15 cells were transfected with different forms of reporter donors. BC: Blank control, BC group is no treatment cells. EGFP control: cells transfected with EGFP-N2 plasmid were as positive control. Reporter plasmid: circular reporter plasmid only. Reporter ^EcoRI^: reporter was digested with EcoRI. Reporter ^EcoRI+HindIII^: reporter was digested with EcoRI and HindIII. Scale bar: 50 μm.

To explore the relationship of fragments around the HAs in the reporter plasmid and unexpected EGFP expression, we also constructed different reporter forms by removing different fragments of the reporter plasmid. For Reporter ^EcoRI^, reporter plasmid was digested with EcoRI and the fragments before the HA-2A-EGFP-Neo-HA were removed. For Reporter ^EcoRI + HindIII^, reporter plasmid was digested with EcoRI and HindIII, removing all the fragments around the HA-2A-EGFP-Neo-HA (Figure 2A and Figure S1B). 

After transfection with different reporter forms, EGFP expression were observed at both PFF and PK15 cells, in which the expression levels were distinct for different cells and different forms. For PFF cells, the EGFP expression with the circular reporter plasmid was 0.57%, which is higher than the cells with Reporter ^EcoRI^ and Reporter ^EcoRI + HindIII^ (Figure 2B). By contrast, PK15 cells expressed lower EGFP (0.21–0.27%) and there was little difference among different reporter forms (Figure 2C). These data suggested EGFP in different forms of the promoterless EGFP reporter could be expressed in the absence of the Cas9/sgRNA. Besides, a similar phenomenon was also found when the promoterless EGFP reporter targeting endogenous *ROSA26* locus [20] was transfected into PFF cells alone (Figure S3).

### 3.3. Higher EGFP Expression in Non-Porcine Cells Transfected with the Porcine Promoterless EGFP Donor Targeting GAPDH

To further study the unexpected EGFP expression in the promoterless EGFP donor and verify its accuracy for accessing HDR, non-porcine cells (CHO-K1, HepG2, and HepaRG cells) were applied and transfected with the constructed promoterless EGFP donor targeting porcine *GAPDH*, in which HDR events could not happen reasonably. Surprisingly, 48 h post-transfection all the non-porcine cells exhibited significantly higher EGFP expression than that in porcine cells.

**Figure 3:**
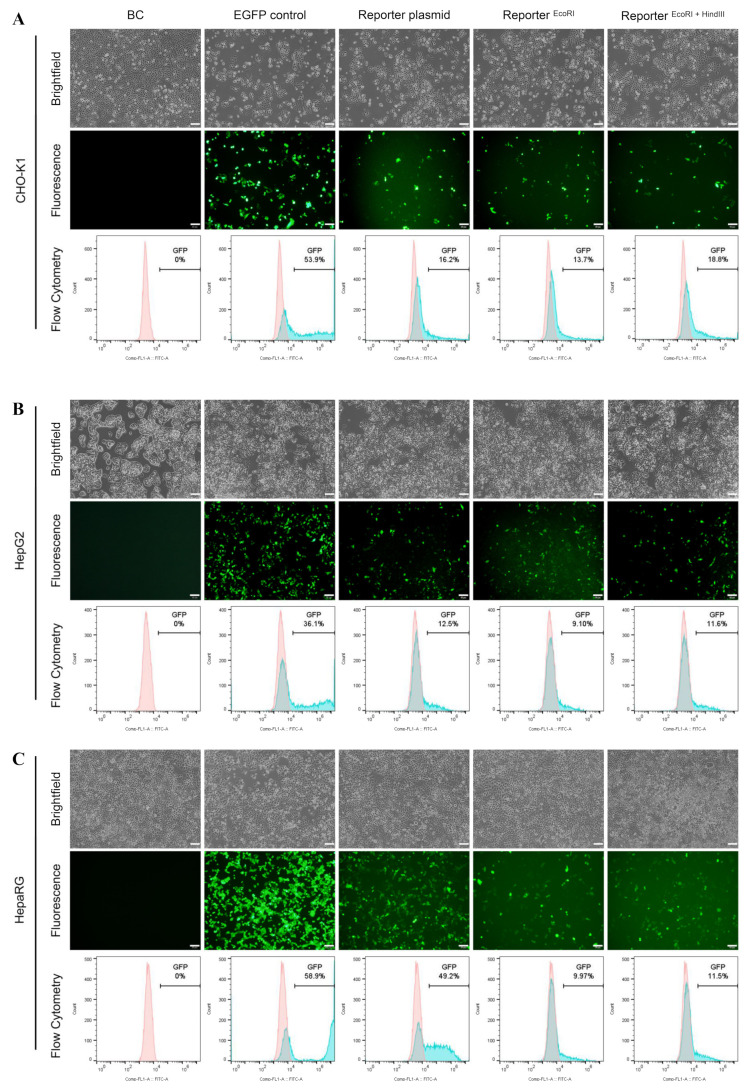
Validation the reporter in non-porcine cells. Fluorescence microscopy and flowcytometry analysis of cells transfected with different forms of reporter at 48 h post-transfection, which involves in (**A**) CHO-K1, (**B**) HepG2 and (**C**) HepaRG. Scale bar: 50 μm.

For CHO-K1 and HepG2 cells, the frequencies of EGFP-positive cells were high about 9.10–18.8%, with no significance among different reporter forms (Figure 3A,B). Even higher, up to 49.4%, EGFP-positive cells appeared in HepaRG cells with circular reporter plasmid, which was comparable with that in the EGFP-N2 positive control group (Figure 3C). Compared with the EGFP expression levels in porcine cells, EGFP expression levels in non-porcine cell lines increased significantly with the same reporter (many times than that in porcine cells), though without ideal homologous region in the genome. Meanwhile, there were also numerous human transcription factors binding sites in the porcine HAs predicted by PROMO database (Figure S4). 

### 3.4. No Precise Insertion but Random Integration in the EGFP Positive Cells

**Figure 4:**
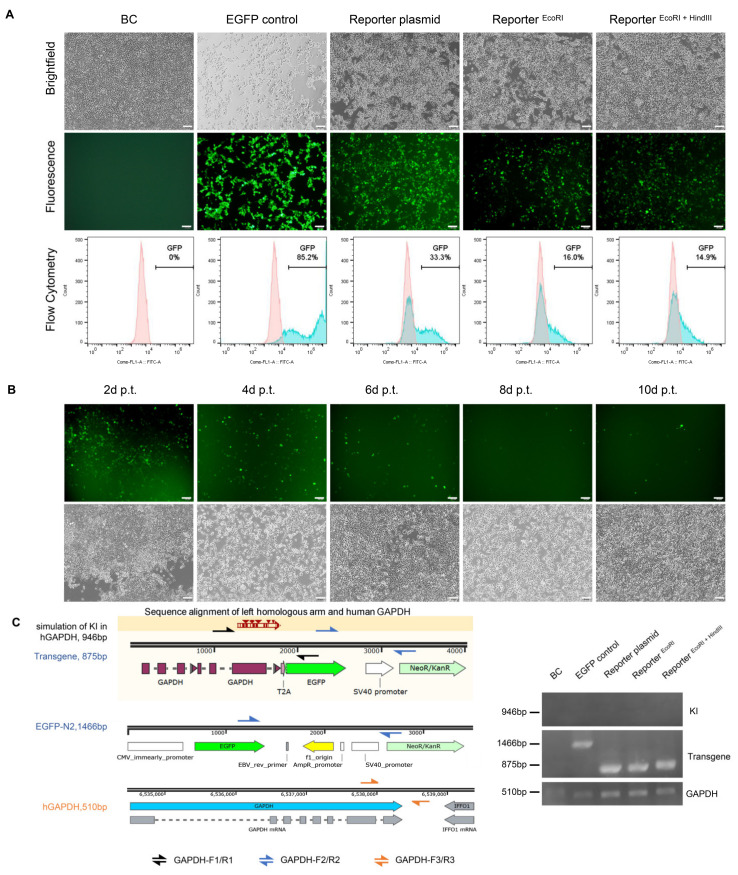
Validation the reporter plasmid’s specificity in 293T. (**A**) 293T cells was transfected with different reporter as described in Figure 2. Scale bar: 50 μm. (**B**) Fluorescence observation of 293T transfected with reporter digested with EcoRI and HindIII every other two days. p.t. stands for post transfection. Scale bar: 50 μm. (**C**) Schematic of PCR primer designed for precise KI and transgene identification. The precise KI primer was used to validate the hypothetical reporter precise KI in human *GAPDH*. Transgene primer was designed within the cargo genes, that were EGFP and *NeoR*. The amplified products of forward primers from exon 8 and reverse primers from exon 9 of human *GAPDH* locus were taken as control.

**Figure 5:**
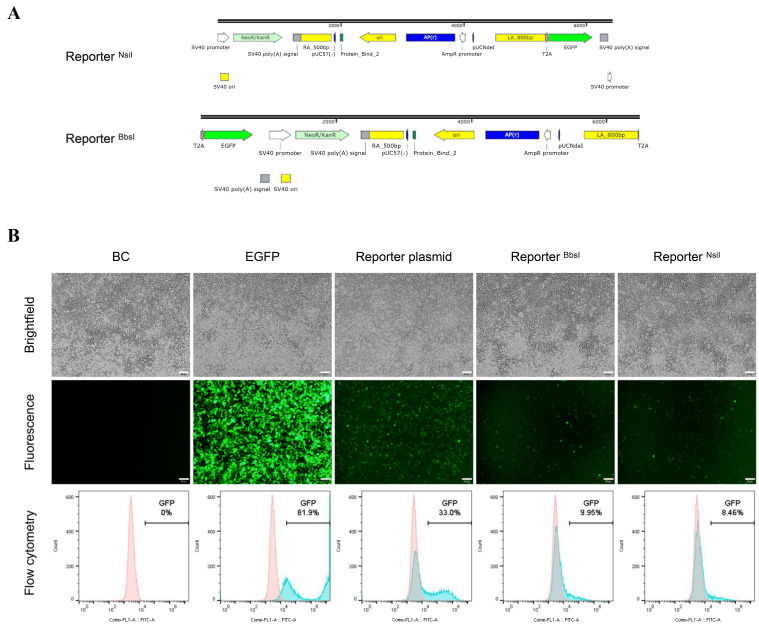
Validation the EGFP expression in 293T cells transfected with different forms of reporter. (**A**) Structure of the reporter digested with NsiI, BbsI, respectively. (**B**) Fluorescence microscopy and flowcytometry analysis of 293T cells transfected with different forms of reporter at 48 h post-transfection. Reporter plasmid, reporter digested with BbsI (Reporter ^BbsI^) or reporter digested with NsiI (Reporter ^NsiI^) were transfected into 293T cells, respectively. Scale bar: 50 μm.

To further explore the relationship between HDR events and fluorescence intensity, we performed a parallel set of experiments in 293T cells (Figure 4A) and EGFP expression continued for 10 days after transfection in 293T cells (Figure 4B). Approximately up to 33% of the cells were EGFP-positive 48 h after the circular reporter plasmid was transfected, a relative lower expression (14.9% and 16.0%) with other reporter forms (Reporter ^EcoRI^ and Reporter ^EcoRI + HindIII^). Oddly enough, when left HAs or right HAs in the reporter donor were separated with the EGFP CDS (Reporter ^BbsI^ and Reporter ^NsiI^) (Figure 5A), the EGFP positive cells still were found after transfection in 293T cells (Figure 5B). The similar phenomenon also appeared in other cells (Figures S5 and S6).

To further identify the HDR events among the GFP positive cells, multiple PCR reactions were performed. The results indicated that no knock-in events happened among all the EGFP positive cells. In contrast, transgene events mediated by random integration occurred in all the EGFP positive cells (Figure 4C). The discovery of no precise insertion, but random integration in the EGFP positive cells, was further demonstrated in molecular levels. Therefore, we suspect that EGFP expression will be too low a resolution to distinguish the occurrence of HDR events from random transgene integration when introducing the promoterless EGFP reporter into cells.

## 4. Discussion

CRISPR/Cas9 mediated-HDR has been extensively used to introduce precise genetic modifications, such as insertions or replacements by HDR with exogenous targeting vectors [35]. To explore strategies of increasing the efficiency of Cas9-mediated HDR, promoterless EGFP reporter constructs are often used to conveniently detect HDR events through fluorescence expression. However, herein, we found data that question the reliability of the promoterless EGFP reporter system.

First, EGFP was expressed at different levels in various cell lines transfected with the promoterless EGFP reporter alone, which would make it a low-resolution reporter for measuring Cas9-mediated HDR events, especially in cells with intrinsically low HDR efficiency. In this study, we found that 48 h following transfection of the promoterless EGFP reporter construct targeting porcine *GAPDH* alone, fluorescence was detectable in both PFF cells and PK15 cells. The expression level was up to 0.57% in PFF cells transfected with the reporter vector alone and not the corresponding Cas9/sgRNA, which indicated that EGFP expression could be driven despite the lack of a promoter. Similar phenomena were observed with other HDR targets and in other cell lines. For example, 3 days after transfecting the promoterless EGFP reporter targeting human *GAPDH* and *ACTB* in 293T cells, fluorescence could be detected by flow cytometry and Western blot [7]. After introducing a similar reporter targeting human *GAPDH* in HCT116 SMMC-7721 and LO2 cells, EGFP fluorescence expression was detectable 5 days after transfection [12,36]. Meanwhile, flow cytometry detected up to 1.81% GFP positive cells when a promoterless GFP reporter targeting *β-actin* was transfected into Neuro2A cells alone [37]. Additionally, cryptic expression of a promoterless EGFP reporter was also observed following injection into mouse zygotes. Overall fluorescence intensities were higher than the bona fide KI efficiencies. Approximately 50% of the EGFP-positive embryos were confirmed to be PCR negative in the most extreme cases [21]. 

Furthermore, expression from the promoterless reporter was further verified when the porcine reporter was transfected into non-porcine cells. Unexpectedly high EGFP expression occurred in HepG2, HepaRG, 293T, and CHO-K1 cells after the porcine reporter was transfected, with up to 33% of 293T cells showing EGFP expression. EGFP expression continued for >10 days. In some cell lines, the high-level of promoterless EGFP expression could exceed HDR efficiency, making it impossible to discriminate HDR events from the more frequent random transgenic integration events. These results indicated that EGFP from the promoterless reporter vector could be expressed to a certain level without HDR events, making it an unreliable reporter for accessing strategies to improve Cas9-mediated HDR.

After performing different experiments to explore the underlying cause for the promoterless EGFP expression, we speculated that various aspects could attribute to the unexpectedly high levels of expression from the promoterless EGFP reporter construct. When HAs of the designed reporter was removed, slightly decreased, but still significant, fluorescence expression was observed in 293T cells, suggesting that the HAs might contain elements that promote EGFP expression. Sequence analysis also indicated numerous transcription factor binding sites that could exist in the HAs of the reporter donor vector targeting porcine *GAPDH* (Supplementary Figure S4). Additionally, EGFP could also be expressed at low levels from the coding sequence itself, even after all other elements around the EGFP sequence were removed. Intriguingly, Mohammadi et al. also recently reported that an EGFP coding sequence could be expressed at up to 50% of the level as CMV-driven cassettes in 293T cells [38]. Furthermore, the established promoterless reporter contains an ATG initiation codon just like those in many published papers [7,12,19,20,21]. After the unexpected EGFP expression was detected, ATG initiation codon is suspect to help the expression. However, the study by Wang et al. indicated the non-specific fluorescence still emerged when the ATG initiation codon was deleted in the promoterless HDR reporter [24]. Therefore, unexpected phenomenon is not only caused by ATG initiation codon, but may be influenced by multiple factors, which need to be further studied. 

In conclusion, our data suggest that the widely used promoterless EGFP reporter construct is a low-resolution HDR screening system. Even in the same 293T cells, Yu et al. reported the efficiency of site-specific insertion of EGFP into *GAPDH* locus was high to 35.7 ± 2.5% [12] while the efficiency reported by Yan et al. was only 1.42% by using the promoterless EGFP reporter [18]. Because of its inaccuracy at reporting HDR events, strategies to increase Cas9-mediated HDR efficiency that have been assessed by such reporters deserve further consideration. In order to overcome the non-specific expression of EGFP, transcription factor binding sites in homologous arms should be predicted before constructing such promoterless reporter. Meanwhile, knock-in editing can be validated by gel electrophoresis and sequencing for a more accurate assessment. There is also now an urgent need to develop a more reliable and high-resolution HDR screening system to better explore strategies of increasing Cas9-mediated HDR efficiency in mammalian cells.

## Data Availability

Not applicable.

## Supplementary Materials

The following supporting information can be downloaded at: https://www.mdpi.com/article/10.3390/cimb44040116/s1, Figure S1: Analysis the constructed reporter plasmid and editing efficiency of the designed sgRNA.; Figure S2: Prediction of indels signatures for the three designed sgRNAs.; Figure S3: Overview of promoterless EGFP reporter targeting the *ROSA26* locus.; Figure S4: Prediction of the transcription factor binding site.; Figure S5: Non-Porcine cells were transfected with different forms of porcine HDR reporter.; Figure S6: Non-Porcine cells were transfected with different forms of porcine HDR reporter.; Table S1: A summary of promoterless reporters targeting endogenous locus for evaluating the CRISPR/Cas9-mediated HDR efficiency.; Table S2: Targeting sequences of CRISPR/Cas9 and oligonucleotides for cloning into sgRNA-expressing vectors.; Table S3: Plasmids made for the study.
