# Supplementary material for: Validation Study to Determine the Accuracy of Widespread Promoterless EGFP Reporter at Assessing CRISPR/Cas9-Mediated Homology Directed Repair
Source: Curr Issues Mol Biol. 2022 Apr 12;44(4):1688–700. doi: 10.3390/cimb44040116 (PMC9164083; doi:10.3390/cimb44040116)
Supplement: Supplementary file 1 [file cimb-44-00116-s001.zip › cimb-1604878-supplementary.pdf]

Supplementary Materials

Supplementary Figures

A

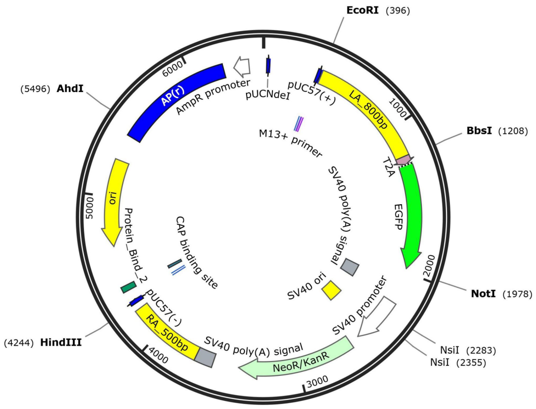

B

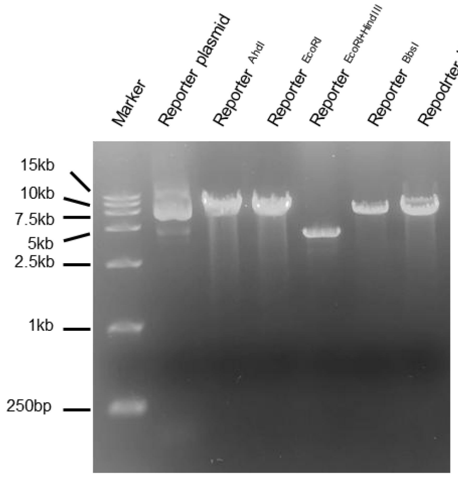

C

Guide sequence + ***PAM***	Specificity	Predicted Efficiency	Off-targets for 0 - 1 – 2 – 3 - 4 mismatches + Next to PAM[a]
CCTCCTCGCGTGCTCTTGCT ***GGG***	91	35	0-0-1-7-84; 0-0-1-1-1
GTGTGTTGGGGGATCGAGTT ***GGG***	96	30	0-0-2-18-183; 0-0-0-0-1
CATGGTCCACATGGCCTCCA ***AGG***	77	54	0-0-6-33-196; 0-0-1-4-9

[a] For each number of mismatches, the number of off-targets is indicated.Off-targets are considered if theyareflanked by oneof these motifs:NGG, NAG, NGA. Shown in red are the off-targets that have no mismatches in the 12 bp adjacent to the PAM. These are the most likely off-targets.

D

sgRNA1: CCTCCTCGCGTGCTCTTGCT **sgRNA2: GTGTGTTGGGGGATCGAGTT**

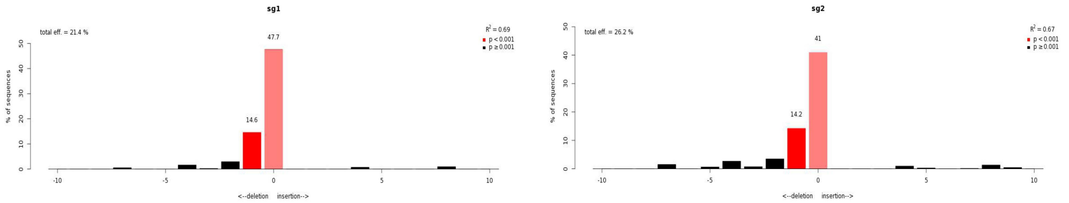

Figure S1. Analysis the constructed reporter plasmid and editing efficiency of the designed sgRNA. (A) Map of the constructed promoterless EGFP reporter. (B) Gel electropherogram of reporter plasmid and corresponding linearized fragments. (C) Three sgRNAs targeting porcine GAPDH locus were designed and their efficiencies and off-targets potency were predicted by CRISPOR in advance. (D) Validation intracellular indels efficiencies and indels patterns of sgRNA1 and sgRNA2 targeting exon 12 of porcine GAPDH by TIDE analysis. Cells were transfected with CRISPR/Cas9 system and harvested after 48 hours for Sanger sequence of GAPDH locus.

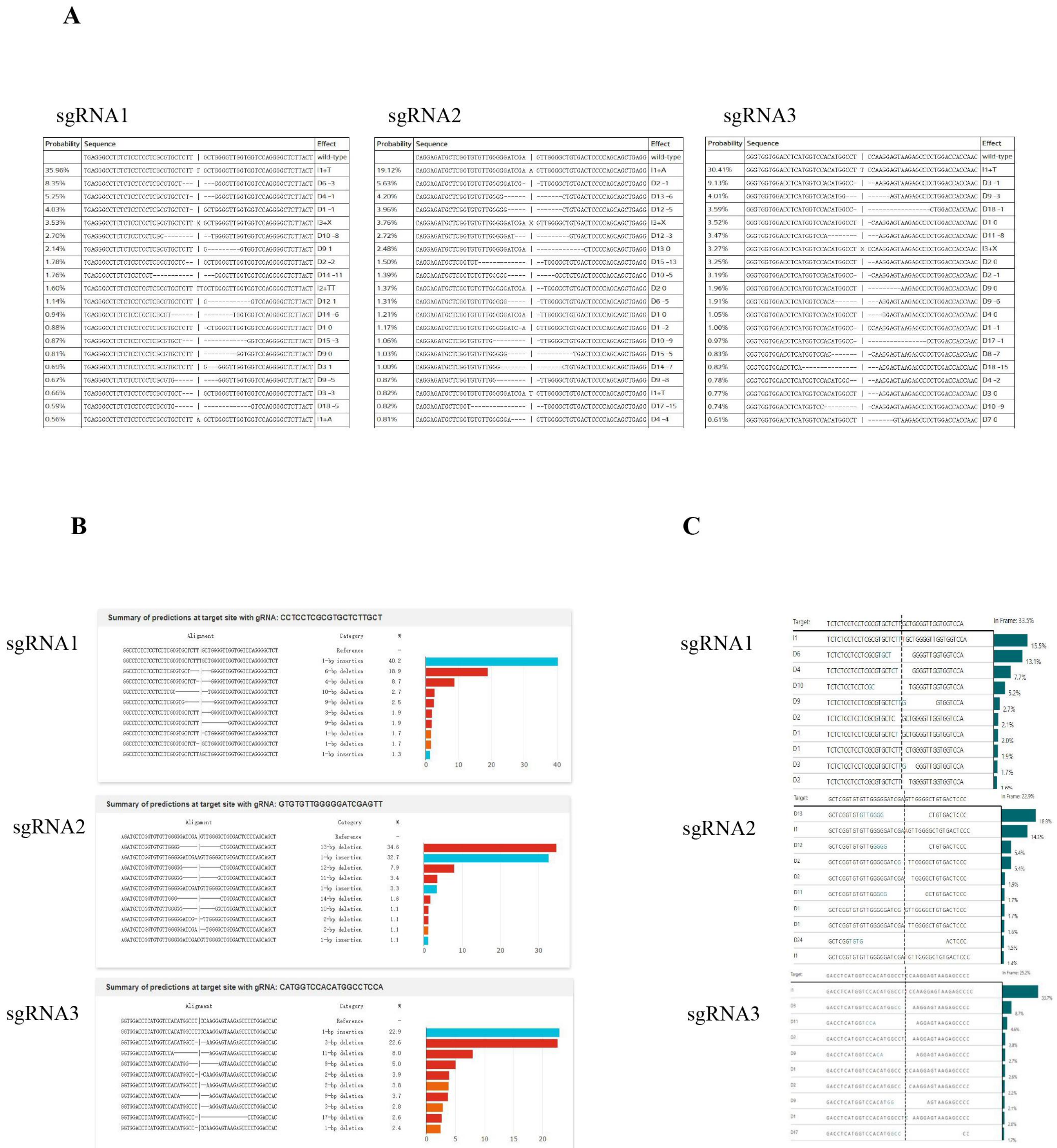

Figure S2. Prediction of indels signatures for the three designed sgRNAs. **(A)** Lindel; **(B)** inDelphi; **(C)** ForeCast.

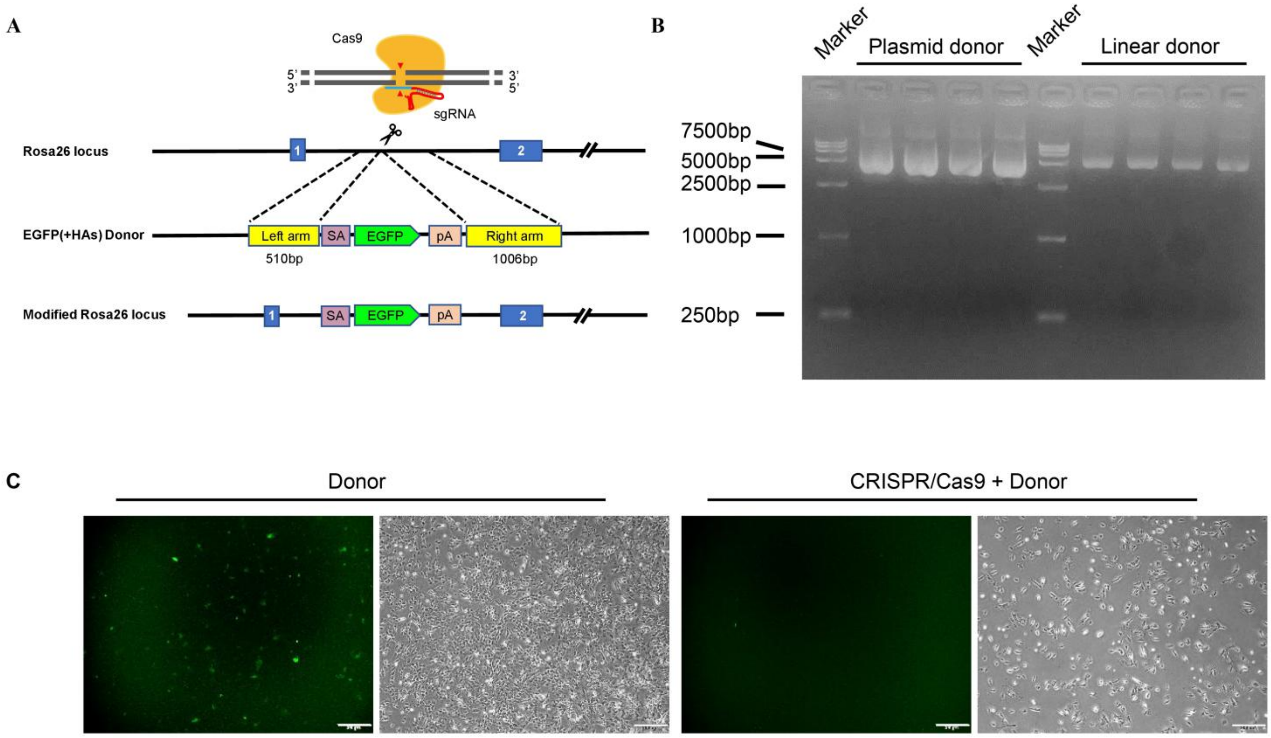

Figure S3. Overview of promoterless EGFP reporter targeting the ROSA26 locus. (A) Schematic of ROSA26 targeted HDR reporter strategy. Donor vector was designed containing a 510-bp left homologous arm and a 1006-bp right homologous arm. SA, splice acceptor; PA, polyA; (B) Gel electropherogram for intact reporter and linearized fragments. (C) Fluorescence microscope of PFF cells 48 hours post transfection of CRISPR/cas9 and HDR reporter donor or reporter donor only. EGFP positive cells can be observed in both groups. Scale bar: 200 μm.

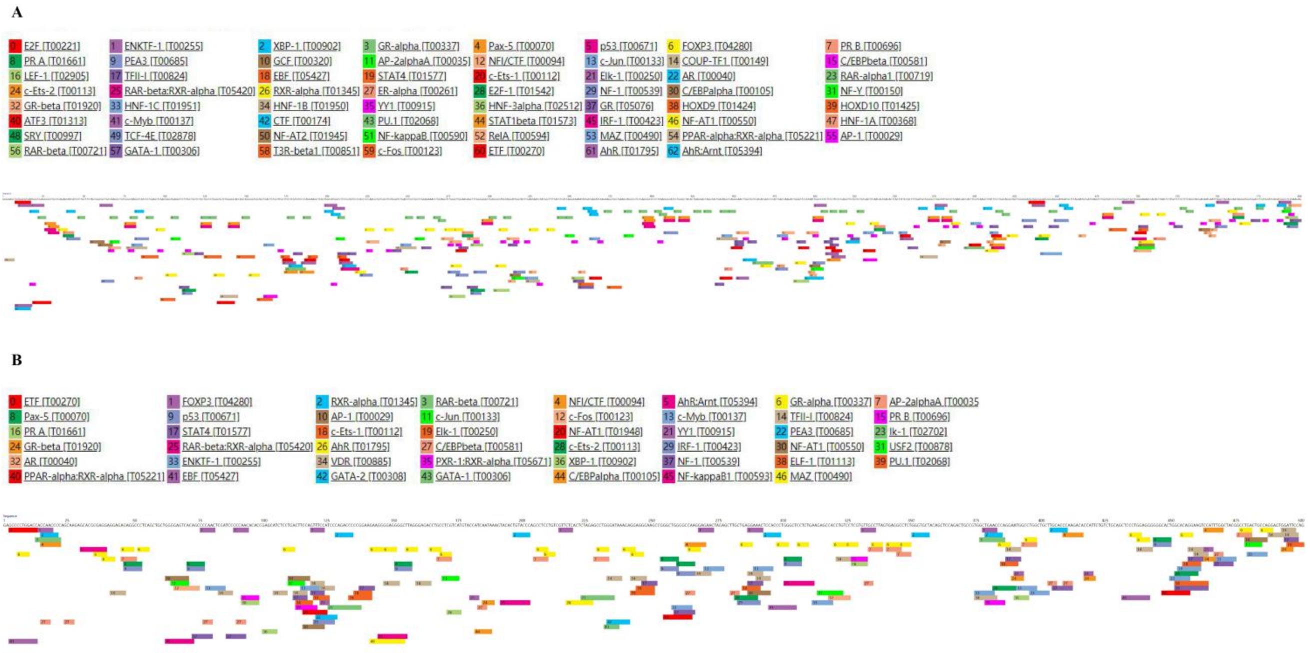

Figure S4. Prediction of the transcription factor binding site, promoter and initiation codons. **(A)** Human transcription factors and its corresponding binding sites in the porcine left homologous arms were predicted by PROMO. Factors predicted within a dissimilarity margin less or equal than 15%. **(B)** Human transcription factors and its corresponding binding sites in porcine right homologous arms were predicted by PROMO. Factors predicted within a dissimilarity margin less or equal than 15%. Interesting transcription factor binding sites of *Sus scrofa* and *Hamster* were not predicted but could still exist due to few data referring to them at present.

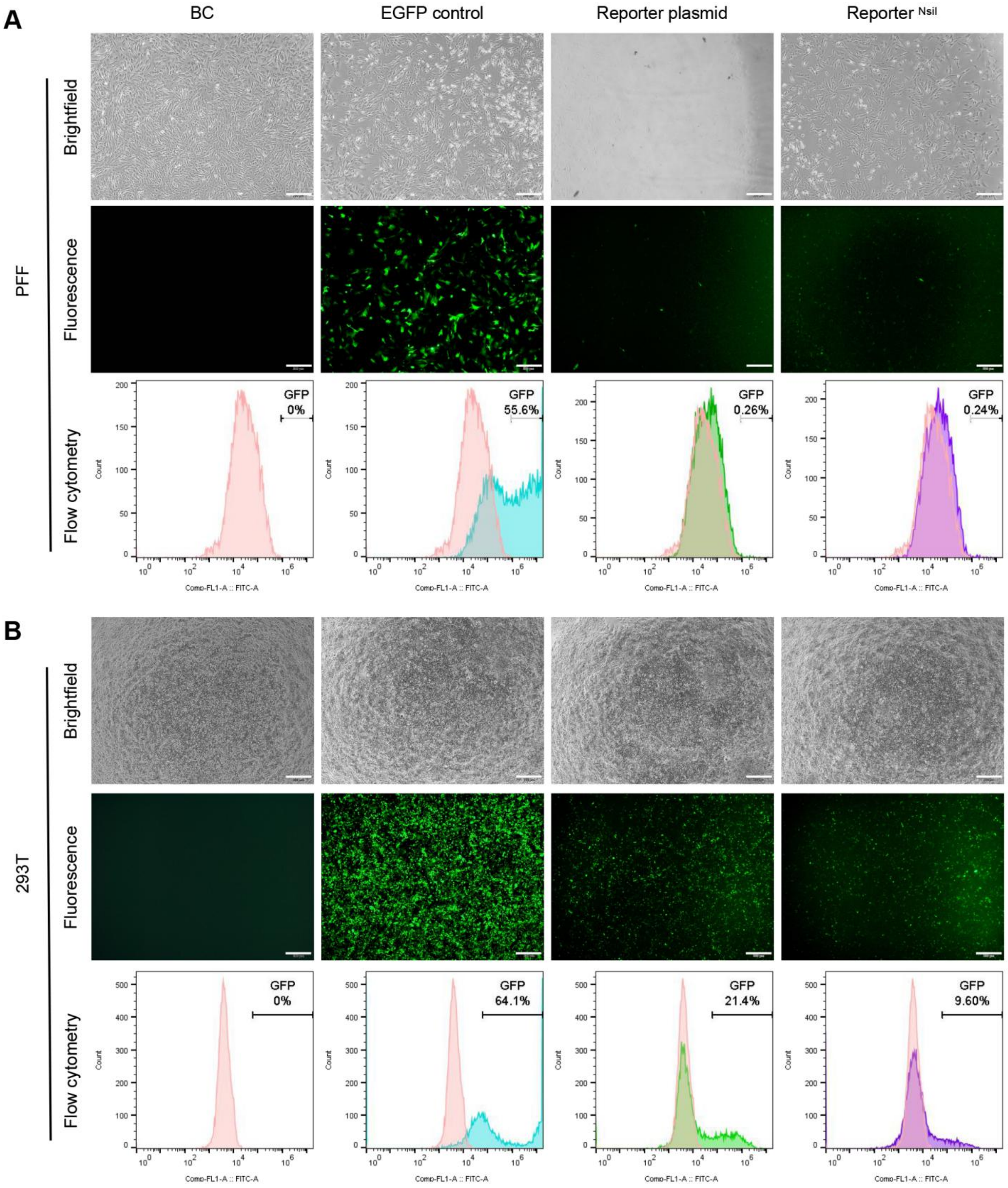

Figure S5. Non-Porcine cells were transfected with different forms of porcine HDR reporter. (A) PFF cells and (B) 293T cells were transfected with different forms of reporter. Blank control: no treatment. EGFP control: transfected with EGFP-N2 plasmid as positive control. Reporter plasmid: reporter donor only. Reporter ^{NsiI}: reporter was digested with NsiI. EGFP fluorescence were detected using fluorescence microscopy and measured by flowcytometry at 2 days post transfection. Scale bar: 200 µm.

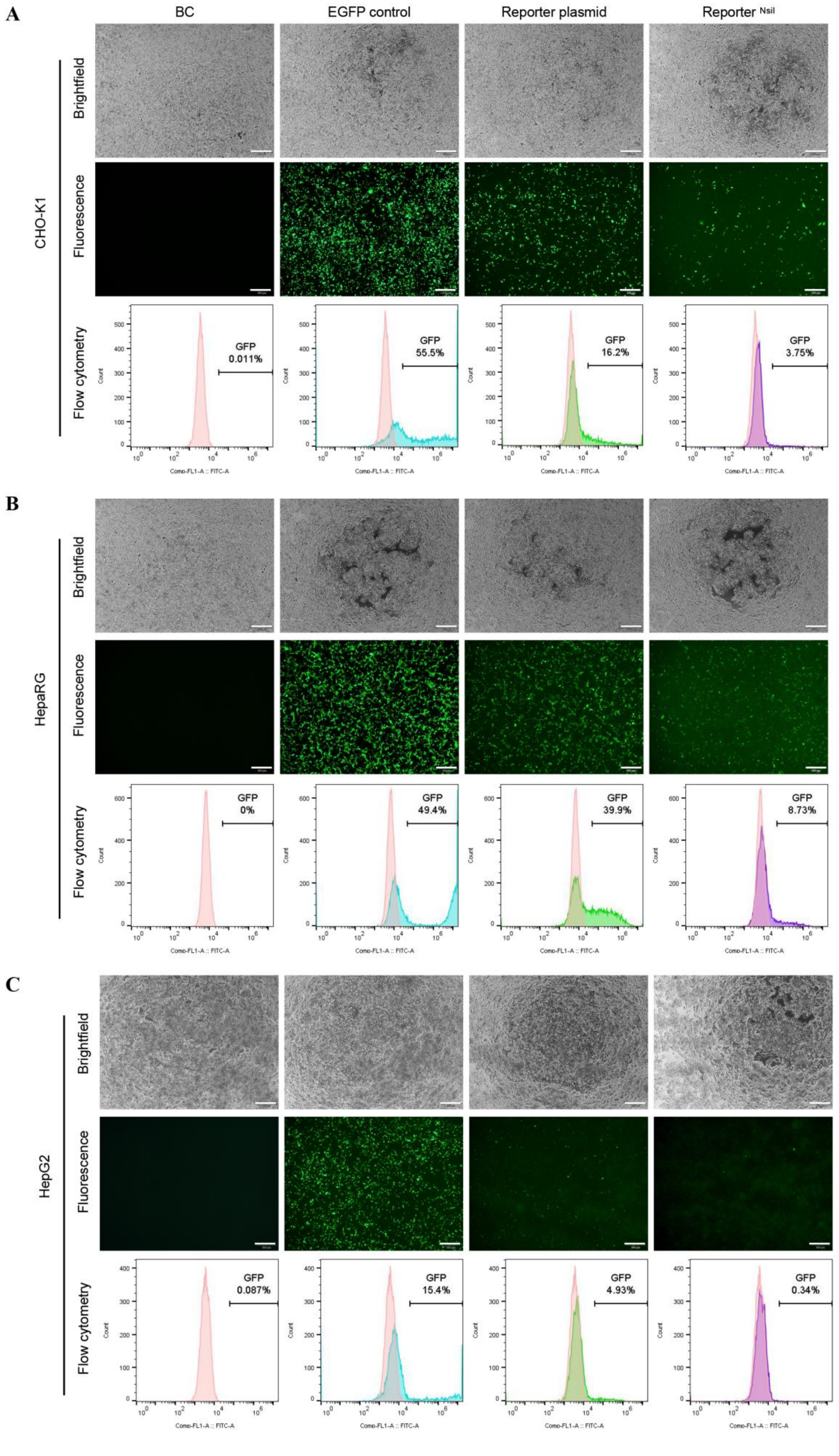
A
BC
EGFP control
Reporter plasmid
Reporter NsiI
CHO-K1
Brightfield
Fluorescence
Flow cytometry
GFP 0.011%
GFP 55.5%
GFP 16.2%
GFP 3.75%
B
HepaRG
GFP 0%
GFP 49.4%
GFP 39.9%
GFP 8.73%
C
HepG2
GFP 0.087%
GFP 15.4%
GFP 4.93%
GFP 0.34%
Count
Comp-FL1-A :: FITC-A

Figure S6. Non-Porcine cells were transfected with different forms of porcine HDR reporter. **(A)** CHO-K1 cells **(B)** HepaRG cells and **(C)** HepG2 cells were transfected with different forms of reporter. Blank control: no treatment. EGFP control: transfected with EGFP-N2 plasmid as positive control. Reporter plasmid: reporter donor only. Reporter^{NsiI}: reporter was digested with NsiI. EGFP fluorescence were detected using fluorescence microscopy and measured by flowcytometry at 2 days post transfection. Scale bar: 200 μm

Supplementary Tables

Table S1: A summary of promoterless reporters targeting endogenous locus for evaluating the CRISPR/Cas9-mediated HDR efficiency

**Promoterless Reporter**	**Conclusion**	**Reference**
Reporter contains internal ribosomalentry site, EGFP sequence with 50-base-pair left and right homology arms (HA). 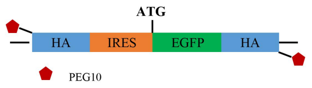	Adding 5′ C6-PEG10 modifications to this promoterless reporter led to up to a five-fold increase in gene knock-in rates at various genomic loci in human cancer and stem cells. For GAPDH locus, the KI rate after applying 5′ C6-PEG10 modifications reporter with short HA (50 bp) was increased from 35.7 ± 2.5% to 59.4 ± 0.50% in HEK293T.	Yu, et al. Nat Chem Biol. 2020
Reporter contains a 200-bp left homology arm, 800-bp rigTF-binding motifs ht homology arm and a T2A–EGFP tag 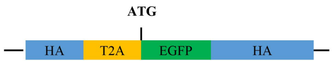	Constructed a fusion protein between Cas9 and TF DNA-binding domain and appended the TF-recognized DNA sequence to the ends of reporter to co-localize the two components in DNA repair process. Based on this stragety, HDR efficiency increase by 2 to 6 fold at various genome sites in human cells. The HDR efficiency at GAPDH in HEK293T was incrased from 9.16% to 16.58%. Adding small-molecule HDR enhancer further increase HDR efficiency to approximately 40%.	Li, et al. J Biol Chem. 2021
Reporter contains 800 bp left and right homologous arms and a P2A-EGFP gene 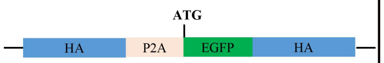	When SHROOM1 siRNA were applied.,HDR efficiency increase from (2.19% ± 0.23%) to (6.66% ± 0.31%) utlizing a double strand PCR reporter at FBLlocus in HEK293T.	Zhao, et al. Int J Mol Sci. 2020
Reporter contains splice acceptor sequences and EGFP gene with different lengths HA (500bp-1500bp). 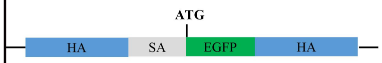	Reporters with different HA lengths resulted in 4.21%-10.61% KI efficiency at ROSA26 in porcine foetal fibroblasts. Synchronization with Nocodazole treatment futher resulted in a 2.8-fold increase in HDR efficiency (29.6%).	Xie, et al. Sci Rep. 2017
Reporter carry a promoterless gene trap cassette with an H2B-EGFP cDNA flanked by homology arms 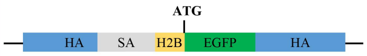	Compared with sgRNA with the Cas9 protein, CRISPR RNA (crRNA)-trans-activating crRNA (tracrRNA)-Cas9 protein complexes led to a 10 fold increase in HDR efficiency at ROSA26 locus in mouse zygotes.	Abe, et al. Cell Rep. 2020
Reporter containes homologous arms and a T2A-EGFP sequence 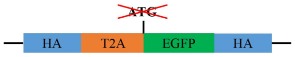	Chromatin reporter led to 2.3-to-7.4 fold HDR increase compared with naked DNA reporter at the tested endogenous loci in human cells (MCF10A, HeLa). HDR efficiency increase from 2.6% to 6% at GAPDH locus in Hela cells.	Cruz-Becerra, et al. Elife. 2020
Reporter contains EGFP sequence with short homology arms (0-30bp) 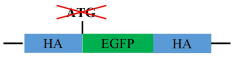	HDR efficiency increase from 0% to 15% when reporters had 0-30 bp HAs targeting Lamin A/C or RAB11 locus in HEK293T cells.	Paix, et al. Proc Natl Acad Sci USA. 2017
Reporters contains P2A–mKate sequences with different length of homologous arm (10-300bp) 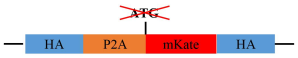	dCas9–SSAP editor (couple microbial single-strand annealing proteins (SSAPs) with catalytically inactive dCas9) has low on-target errors and minimal off-target effects, showing higher accuracy than canonical Cas9 methods. Reporters with different HA lengths resulted in 2%-5% KI efficiency at DYNLT1 locus in HEK 293T cells.	Wang, et al. Nat Cell Biol. 2022
Reporters contains T2A–EGFP sequences with different length of homologous arm (50-1800bp) 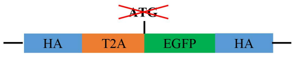	Co-transfection of the HDR-USR(universal surrogate reporter) system into host cells and transient puromycin selection efficiently achieves enrichment of HDR-modified cells. 2.3-to-7.4 fold enrich with reporter using 50 bp-to-1800 bp homologous arms at GAPDH in HEK293T cells (HDR efficiency increase from 1.42% to 9.6%).	Yan, et al. Mol Ther Nucleic Acids. 2020

Table S2: Targeting sequences of CRISPR/Cas9 and oligonucleotides for cloning into sgRNA-expressing vectors

Target gene	Target sequence with PAM	sgRNA name	Forward oligonucleotides for cloning	Reverse oligonucleotides for cloning	Vector	Restriction enzyme
GAPDH (Sus scrofa)	CCTCCTCGCGTGCTCTTGCT*GGG*	sgRNA1	caccCCTCCTCGCGTGCTCTTGCT	aaacAGCAAGAGCACGCGAGGAGG	pU6-sgRNA-vector	BbsI
GAPDH (Sus scrofa)	GTGTGTTGGGGGATCGAGTT*GGG*	sgRNA2	caccGTGTGTTGGGGGATCGAGTT	aaacAACTCGATCCCCCAACACAC	pU6-sgRNA-vector	BbsI
GAPDH (Sus scrofa)	CATGGTCCACATGGCCTCCA*AGG*	sgRNA3	caccCATGGTCCACATGGCCTCCA	aaacTGGAGGCCATGTGGACCATG	pU6-sgRNA-vector	BbsI

Table S3: pU6-sgRNAplasmids made for the study

No.	Name	Purpose	Generation strategy
1	pU6 -sgRNA1 expression vector	Plasmid expressing single guide RNA targeting Sus scrofa *GAPDH*	The annealed product of oligonucleotides (sgRNA1, 5'-caccCCTCCTCGCGTGCTCTTGCT-3' and 5'-aaacAGCAAGAGCACGCGAGGAGG-3') were annealed to form double -strand DNA and then cloned into BbsI restriction enzyme-digested U6-sgRNA cloning vector.
2	pU6 -sgRNA2 expression vector	Plasmid expressing single guide RNA targeting Sus scrofa *GAPDH*	The annealed product of oligonucleotides (sgRNA2, 5'-caccGTGTGTTGGGGGATCGAGTT-3' and 5'-aaacAACTCGATCCCCCAACACAC-3') were annealed to form double -strand DNA and then cloned into BbsI restriction enzyme-digested U6-sgRNA cloning vector.
3	pU6 -sgRNA3 expression vector	Plasmid expressing single guide RNA targeting Sus scrofa *GAPDH*	The annealed product of oligonucleotides (sgRNA3, 5'-caccCATGGTCCACATGGCCTCCA-3' and 5'-aaacTGGAGGCCATGTGGACCATG-3') were annealed to form double -strand DNA and then cloned into BbsI restriction enzyme-digested U6-sgRNA cloning vector.
